# Inhibition of stress-inducible HSP70 impairs mitochondrial proteostasis and function

**DOI:** 10.18632/oncotarget.17321

**Published:** 2017-04-21

**Authors:** Julia I-Ju Leu, Thibaut Barnoud, Gao Zhang, Tian Tian, Zhi Wei, Meenhard Herlyn, Maureen E. Murphy, Donna L. George

**Affiliations:** ^1^ Department of Genetics, The Raymond and Ruth Perelman School of Medicine at the University of Pennsylvania, Philadelphia, PA 19104, USA; ^2^ Melanoma Research Center and Program in Molecular and Cellular Oncogenesis, The Wistar Institute, Philadelphia, PA 19104, USA; ^3^ Department of Computer Science, New Jersey Institute for Technology, Newark, NJ 07102, USA

**Keywords:** HSP70, chaperone, mitochondria, proteostasis, PABPC1

## Abstract

Protein quality control is an important component of survival for all cells. The use of proteasome inhibitors for cancer therapy derives from the fact that tumor cells generally exhibit greater levels of proteotoxic stress than do normal cells, and thus cancer cells tend to be more sensitive to proteasome inhibition. However, this approach has been limited in some cases by toxicity to normal cells. Recently, the concept of inhibiting proteostasis in organelles for cancer therapy has been advanced, in part because it is predicted to have reduced toxicity for normal cells. Here we demonstrate that a fraction of the major stress-induced chaperone HSP70 (also called HSPA1A or HSP72, but hereafter HSP70) is abundantly present in mitochondria of tumor cells, but is expressed at quite low or undetectable levels in mitochondria of most normal tissues and non-tumor cell lines. We show that treatment of tumor cells with HSP70 inhibitors causes a marked change in mitochondrial protein quality control, loss of mitochondrial membrane potential, reduced oxygen consumption rate, and loss of ATP production. We identify several nuclear-encoded mitochondrial proteins, including polyadenylate binding protein-1 (PABPC1), which exhibit decreased abundance in mitochondria following treatment with HSP70 inhibitors. We also show that targeting HSP70 function leads to reduced levels of several mitochondrial-encoded RNA species that encode components of the electron transport chain. Our data indicate that small molecule inhibitors of HSP70 represent a new class of organelle proteostasis inhibitors that impair mitochondrial function in cancer cells, and therefore constitute novel therapeutics.

## INTRODUCTION

Mitochondria have attracted cancer researchers since the 1950s, when the differences in metabolism between transformed and normal cells were first described by Warburg [1–3]. Interest in mitochondria as a cancer target accelerated when it was discovered that this organelle is critical for the intrinsic apoptosis pathway. This information led to the development of a number of compounds that induce the mitochondrial pathway of cell death, such as inhibitors of the BCL2 family of anti-apoptotic proteins [4, 5]. More recently, mitochondrial oxidative phosphorylation was shown to be required for transformation by oncogenic KRAS, and to play a role in tumor metastasis, stemness, and drug resistance, as well as poorer cancer survival [6–13]. Consequently, compounds have been sought that selectively or preferentially target mitochondrial function in tumor cells. One therapeutic approach focuses on the identification of inhibitors of mitochondrial protein quality control. With the discovery that the molecular chaperones HSP90 and the closely related protein TRAP-1 locate to the mitochondria of tumor cells, but not most normal cells, the Altieri group pioneered the development of a series of mitochondria-targeted HSP90 inhibitors called Gamitrinibs [13–16]. Gamitrinibs selectively target mitochondrial, but not cytosolic, HSP90. These compounds show the ability to disrupt mitochondrial protein quality control, leading to altered tumor bioenergetics and inhibition of tumor progression and metastasis [9, 13–18]. As further proof of concept for this strategy, silencing or inhibition of the mitochondrial protease ClpP, which normally degrades mitochondrial proteins damaged by oxidative stress, was shown to be an effective anti-tumor strategy, and a ClpP inhibitor was shown to delay tumor growth in an animal model of acute myeloid leukemia [19, 20].

The HSP70 chaperones are critical mediators of protein quality control [21–25]. There are more than eight members of this family. Some members are organelle-specific (GRP78 in the ER and GRP75 in the mitochondria), while others are constitutively expressed in all tissues and required for the proper folding of newly-translated proteins (HSC70). HSP70 (also called HSPA1A or HSP72) is one of only two family members that are up-regulated by heat shock and other stresses. Consequently, this molecular chaperone is highly expressed in tumor cells, but shows markedly lower, or even undetectable, expression in unstressed normal cells and tissues [21–25]. Unlike other family members, HSP70 is not required for viability, and knockout mice for HSP70 are viable and fertile [26]. However, these mice are more susceptible to a variety of stresses, which is consistent with the predominantly stress-induced role of this chaperone. HSP70 is present at multiple cellular locations, and exhibits a number of important pro-survival functions, including: facilitating the proper folding of polypeptide chains; aiding in the assembly of protein complexes; mediating the transport of certain clients across cell membranes; targeting misfolded/damaged proteins for degradation. The tumor-related expression of HSP70, along with its key role in regulating overall protein quality control in stressed cells, have rendered this chaperone an attractive target for cancer therapy [21–25].

We previously demonstrated that the small molecule 2-phenylethynesulfonamide (PES, also called pifithrin-mu) and the PES-derivative triphenyl(phenylethynyl)phosphonium bromide (PET-16) directly and selectively bind to the C-terminus of HSP70 [27–29]. Both of these compounds inhibit the chaperone activity of HSP70, and they are preferentially toxic to tumor cells compared to normal or non-transformed cells[27–31]. HSP70 inhibition affects proteins and signaling pathways that function in several cellular locations, including cytosol, lysosome and nucleus. We have shown that our novel series of phenylethyne-based HSP70 inhibitors is effective in pre-clinical models of melanoma and B cell lymphoma and that they inhibit tumor metastasis. For example, these inhibitors retain cytotoxicity in cultured melanomas resistant to BRAF inhibitors. Also, when tested in mice with melanoma xenografts, PET-16 enhances the durability of response to BRAF inhibition [27, 28, 31]. Mechanistically, we and others have shown that the cytotoxic effects of these HSP70 inhibitors generally do not associate with enhanced apoptosis; rather they lead to impaired protein quality control, including abrogated autophagy, decreased proteasome function and an accumulation of aggregates of insoluble or denatured proteins [27–36].

The potential efficacy of HSP70 inhibition for disrupting mitochondrial protein quality control and function had not previously been explored. In addressing that issue, we now find that a significant fraction of endogenous HSP70 protein is constitutively present at the mitochondria of tumor cells but is generally undetectable or present at quite low levels in mitochondria of normal and non-transformed cells. Treatment of tumor cells with HSP70 inhibitors leads to a loss of mitochondrial membrane potential and also produces an alteration in the abundance, and/or the expression pattern, of several mitochondrial proteins and RNA species. These results indicate that disrupting the HSP70-based chaperone machinery promotes mitochondrial dysfunction and represents a novel approach for targeting the mitochondria of tumor cells.

## RESULTS

### Tumor cell mitochondria contain stress-inducible HSP70

**Figure 1:**
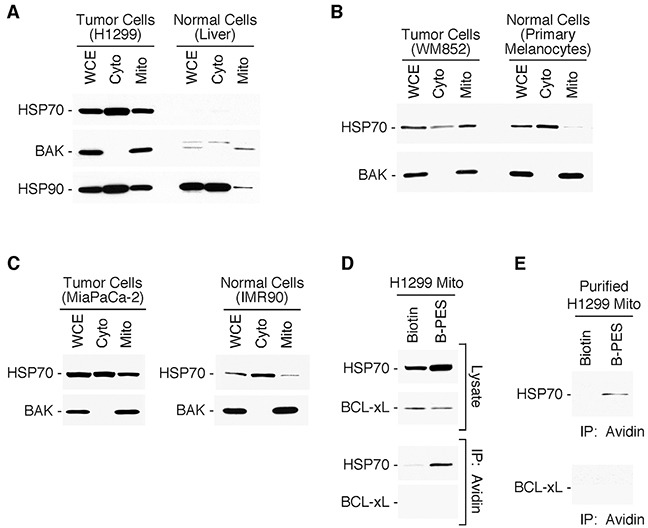
Stress-inducible HSP70 is present at mitochondria of tumor cells **(A-C)** Whole cell (WCE), cytosolic (Cyto) and mitochondrial (Mito) protein extracts were prepared from tumor cell lines H1299 lung carcinoma, MiaPaCa-2 pancreatic cancer or WM852 melanoma cells; from normal cells (mouse liver); and from cultures of primary human melanocytes and immortalized human lung fibroblasts IMR90. Western blots of the proteins were probed as indicated. For each set of extracts, equal amount of protein was loaded. **(D-E)** H1299 cells or isolated mitochondria, respectively, were treated with Biotin or Biotin-tagged PES (B-PES). Mitochondrial extracts were prepared, and interacting proteins captured using NeutrAvidin agarose resins. After eluting from the resin, PES-associated proteins were immunoblotted using the indicated antibodies.

We previously performed a proteomic analysis of highly purified mitochondria isolated from tumor cells on sucrose gradients, and noted that a portion of the major cytosolic stress-induced form of HSP70 appeared to co-purify with mitochondria [37]. To extend this finding, we analyzed whole cell, cytosolic, and mitochondrial fractions from different tumor cell lines for the presence of HSP70, using an antibody that is specific for the major stress-induced form of this protein (also called HSP72 or HSPA1A). We detected an abundant pool of HSP70 co-purifying with the cytosol and mitochondria in all tumor cells examined (Figure 1A-1C). In contrast, HSP70 was largely undetectable in cytosol and mitochondria purified normal mouse liver (Figure 1A). The latter observation corroborates the findings of our group and others that HSP70 is undetectably expressed in most unstressed normal cells and tissues, but is overexpressed in tumor cells. As noted previously [21], cultured cells generally express higher levels of HSP70 relative to normal tissues and non-cultured cells, a likely consequence of the enhanced physiologic stress associated with the cell culturing process itself. Therefore, we extended our studies to examine HSP70 at mitochondria in cultures of primary human melanocytes as well as in the non-transformed human lung fibroblast cell line, IMR90. HSP70 expression levels in mitochondria of such non-transformed cells are clearly lower than those in the tumor cell lines, even though the non-transformed cells express abundant HSP70 in the cytosol (Figure 1B and 1C). A similar finding was reported for the molecular chaperone HSP90, and also shown here (Figure 1A), supporting the idea that a distinct mitochondrial chaperone network is established in tumor cells to help accommodate the enhanced stress of the tumor microenvironment [13–16].

### PES binds to HSP70 at mitochondria

As we previously reported, the free amide group of PES can be biotinylated (B-PES), and this molecularly tagged molecule maintains the ability to bind and inhibit HSP70 in cells [27, 28, 30]. PES binding is selective, and it does not detectably interact with the mitochondrial (GRP75)- or ER-resident (GRP78)- members of the HSP70 family [27, 30]. Therefore, we tested whether PES interacts with HSP70 protein localized at mitochondria. Using biotin-tagged PES, we performed two different experiments to address this question. First, we incubated cells with B-PES, and isolated mitochondria from the treated cells. We then immunoprecipitated the tagged PES as well as PES-interacting proteins with NeutrAvidin agarose resins, as we previously described [27, 28, 30]. The results of this study revealed the presence of HSP70 in avidin immunoprecipitates from mitochondria isolated from cells treated with B-PES, but not biotin alone (Figure 1D). Second, we purified mitochondria from tumor cells, and treated these purified mitochondria with B-PES, followed by avidin immunoprecipitation; this analysis also revealed HSP70 in B-PES/avidin immunoprecipitates (Figure 1E), confirming that PES interacts with HSP70 at mitochondria.

### Inhibiting HSP70 impairs mitochondrial membrane potential and ATP production

**Figure 2:**
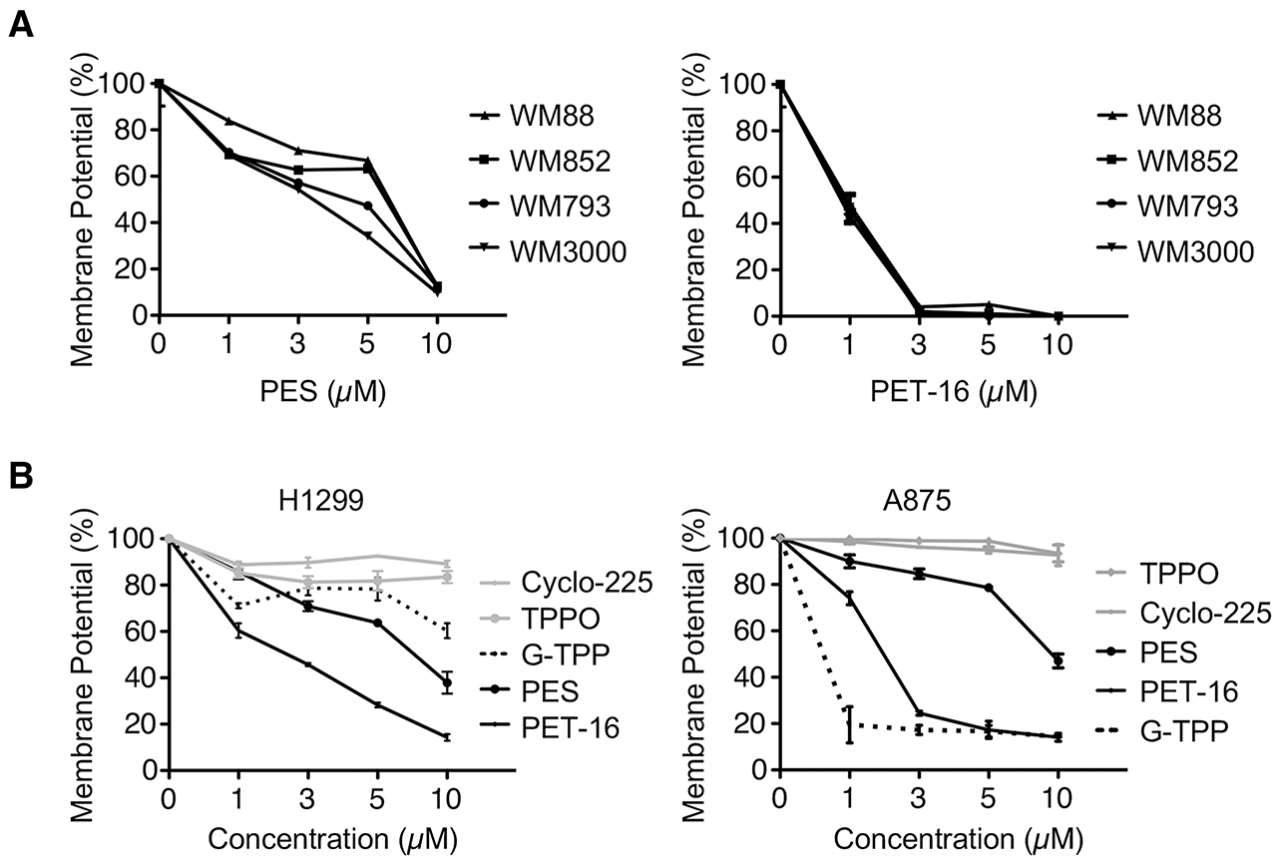
HSP70 inhibitors alter mitochondrial membrane potential **(A-B)** Mitochondrial membrane potential was measured in the cell lines indicated (H1299 lung cancer and melanomas A875, WM88, WM852, WM793, WM3000). Cells were treated with PES, PET-16, G-TPP or structurally related control compounds (TPPO and Cyclo-225). Results shown are the mean ± SD of at least three independent experiments relative to control (DMSO-treated) cells.

We then sought to determine whether treating cells with the HSP70 inhibitors PES and PET-16 would have an impact on mitochondrial function. Of note for this study, PET-16 contains a linked triphenylphosphonium group (TPP), which enhances the targeting of compounds to mitochondria [38, 39]. We treated several different melanoma cell lines with the HSP70 inhibitors PES and PET-16, which led to the appearance of mitochondria exhibiting altered morphology (Supplementary Figure 1). The HSP70 inhibitors also caused a dose-dependent loss of mitochondrial membrane potential (MMP) in all the tumor cells examined, including a series of melanomas (Figure 2A). Also, as illustrated in Figure 2B, the reduced MMP resulting from HSP70 inhibition was similar to that resulting from exposure to Gamitrinib (G-TPP) (Figure 2B), a selective inhibitor of mitochondria-localized HSP90 proteins [13–16]. We also noted that the degree to which both the HSP70 and HSP90 inhibitors disrupt MMP varies somewhat among the different cell lines tested, although the specific molecular basis for these differences remain to be determined. In contrast to the effects of PES and PET-16, the TPPO moiety alone, or an inactive version of PET-16 (Cyclo-225) had no effect on MMP (Figure 2B). The structures of all PES-based compounds and controls are depicted in Supplementary Figure 2.

**Figure 3:**
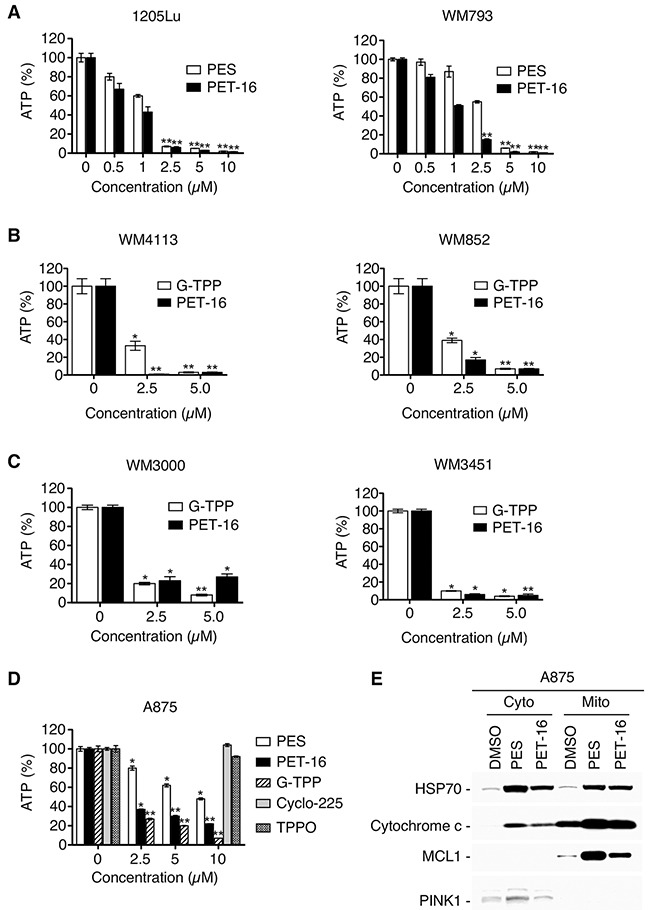
HSP70 inhibitors alter ATP abundance and promote cytochrome c re-distribution **(A-D)** The indicated melanoma tumor cell lines were treated for 48 h with various concentrations of PES, PET-16, G-TPP, Cyclo-225 or TPPO, and cellular ATP abundance was assayed. Results shown are the mean ± SD of at least three independent experiments relative to DMSO-treated control cells (*P<0.05, **P<0.005). **(E)** Western blot analysis for the indicated proteins extracted from mitochondria or cytosolic fractions of A875 cells treated for 24 h with 20 μM PES or PET-16.

Given these results, we assessed the impact of PES and PET-16 on cellular ATP levels. As illustrated (Figure 3A-3D), the HSP70 inhibitors caused a significant decrease in cellular ATP abundance in each of several tumor cell lines examined. In contrast, the inactive derivative Cyclo-225 and the TPPO moiety alone had no discernable effect in these assays (Figure 3D). As noted above, the degree to which PET-16 was able to promote a reduction in ATP varied among the cell lines examined, but was generally comparable to the mitochondrial toxin G-TPP (Figure 3B-3D).

### Inhibiting HSP70 leads to impaired mitochondrial protein quality control

MMP loss associated with a rupture of the outer mitochondrial membrane and release of cytochrome *c* is a feature of mitochondrial permeability transition. Accordingly, we extended these studies to examine the ability of our HSP70 inhibitors to induce cytochrome c release from mitochondria. Both PES and PET-16 induced the appearance of cytochrome c in the cytosolic fraction of treated cells (Figure 3E), indicative of altered mitochondrial integrity. Note also that in the treated cells there is an increase in the abundance of MCL1 (Figure 3E); this mitochondrial protein typically has a very short half-life and is rapidly turned over by proteasome-mediated degradation [40]. Thus, this increase in mitochondrial MCL1 and cytochrome c levels also supports the conclusion that HSP70 inhibition disrupts normal mitochondrial proteostasis.

**Figure 4:**
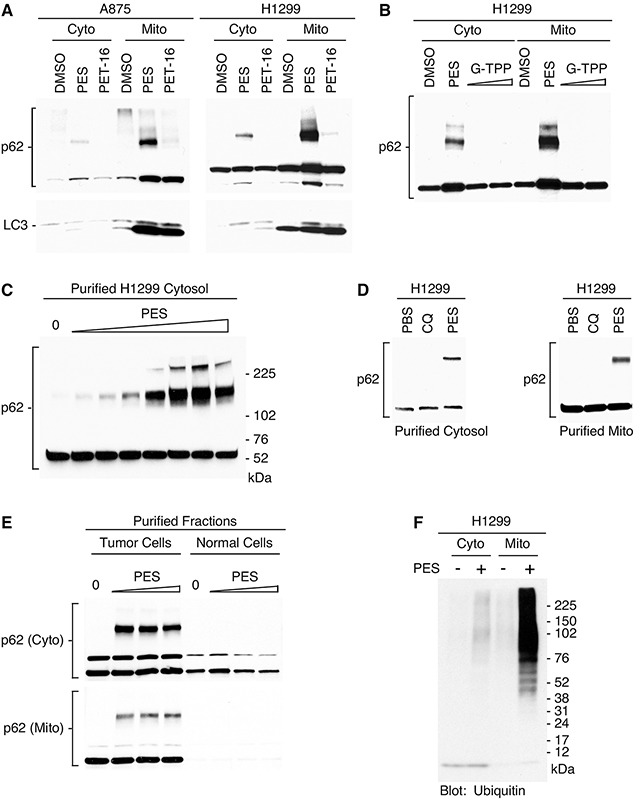
PES interacts with HSP70 at mitochondria and promotes p62 oligomerization **(A)** Western blot analysis of p62 and LC3 protein forms in cytoplasmic and mitochondrial fractions of cells treated with PES or PET-16 (20 μM). **(B)** H1299 tumor cells were treated with PES (20 μM) or G-TPP (2.5 or 10 μM) followed by western blot analysis for expression of p62. **(C)** Purified cytosolic fractions from H1299 tumor cells were treated with increasing amounts of PES (0.5-200 μM) followed by western blot analysis for expression of p62. **(D)** Western blot analysis of p62 in purified cytosolic or mitochondrial fractions of H1299 cells following incubation with 20 μM of chloroquine (CQ) or PES. **(E)** Purified cytosolic or mitochondrial extracts from H1299 tumor cells or normal mouse liver were treated with increasing amounts of PES (0.5-200 μM) followed by western blot analysis for expression of p62. **(F)** Western blot of proteins isolated from cytoplasmic and mitochondrial fractions of PES-treated cells was probed with anti-ubiquitin antibody.

We extended these studies to test if HSP70 inhibition would alter the expression at mitochondria of the autophagy adaptor protein p62^SQSTM1^ (hereafter referred to as p62). This important multifunctional signaling-scaffold protein is present in cytosolic fractions and also localizes to mitochondria under normal physiological conditions, as well as after stress. It plays a key role in maintenance of normal mitochondrial functioning and participates in the triage of damaged proteins and of the organelles themselves [41, 42]. To assess p62 expression, we treated two different tumor cell lines with PES and PET-16, followed by purification of mitochondria and western analysis for p62. The results revealed an accumulation and aggregation of p62, exemplified by an increase in p62 monomers and oligomers co-purifying with mitochondria (Figure 4A). This was accompanied by an increase in the abundance of the lipidated form (LC3-II) of microtubule-protein light chain (LC3) as presented in Figure 4A; LC3-II accumulation is a marker of damaged or impaired mitochondrial [43]. Note that p62 oligomerization is not detectably induced by the mitochondrial HSP90 inhibitor G-TPP (Figure 4B), which generally promotes protein destabilization and subsequent degradation. These results provide additional evidence of enhanced proteotoxic stress and impaired mitochondrial protein quality control resulting from HSP70 inhibition.

We next assessed the impact of PES dose escalation on p62 expression using purified cytosolic extracts. We found that the HSP70 inhibitor promoted the accumulation and oligomerization of p62 in a dose-dependent manner (Figure 4C). This effect was not simply reflective of impaired autophagy since p62 oligomerization was not induced by the lysosome- and autophagy-inhibitor chloroquine (Figure 4D). PES can promote p62 oligomerization even in isolated mitochondria purified from tumor cells, but not those of normal cells (Figure 4E). The latter result is entirely consistent with our previously published findings that PES causes very little p62 oligomerization in primary melanocytes and immortalized, non-transformed cell lines such as IMR90 [28, 30]. Protein aggregates and damaged molecules are tagged with ubiquitin to target them for degradation. p62 associates with ubiquitylated proteins, sequestering this potentially toxic cargo for autophagic degradation. The accumulation of oligomeric forms of p62 therefore suggests a failure in the autophagic/proteasomal clearance of damaged proteins and aggregates. Thus, we analyzed mitochondria purified from PES-treated cells for the accumulation of polyubiquitylated proteins. As revealed by western blot analysis, polyubiquitylated proteins were markedly more abundant in the mitochondria following treatment with the HSP70 inhibitor (Figure 4F). These combined data are consistent with the conclusion that PES and PET-16 impair the function of mitochondrial HSP70, and that this destabilizes mitochondrial protein quality control.

**Figure 5:**
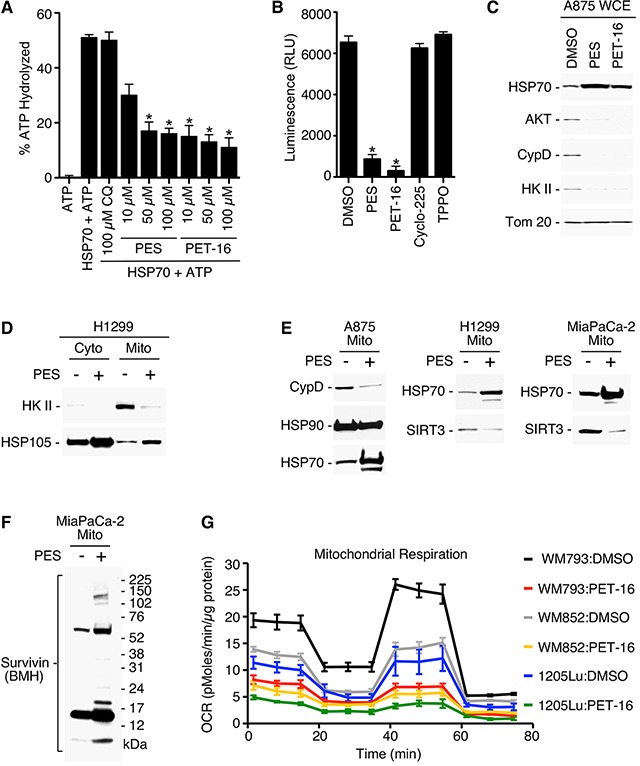
PES and PET-16 alter HSP70 ATPase activity and mitochondrial protein quality control **(A)** Purified full-length HSP70 protein generated in bacteria was incubated with each of the compounds specified and assayed for intrinsic ATPase activity. Results shown are the mean ± SD of at least three independent experiments. *P<0.05. **(B)** A875 human melanoma cell line stably expressing a luciferase expression construct was treated with the indicated compounds and assayed for relative luciferase luminescence (RLU). Each graphical representation indicates the mean ± SD of at least three independent cultures relative to control cells. *P<0.01. **(C)** A875 cells were treated with 20 μM PES or PET-16 as indicated. Cell lysates were fractionated into detergent-soluble preparations and assayed by Western blotting for the proteins indicated. **(D-F)** The cell lines indicated were treated with PES, mitochondrial and/or cytosolic fractions were isolated, and western blots probed for the proteins indicated. **(G)** Mitochondrial oxygen consumption rate (OCR) in the melanoma cell lines indicated was measured before and after treatment with 1 μM PET-16 for 24 h. Each graphical representation indicates the mean ± SD of at least three independent cultures.

We sought to clarify the pathways by which HSP70 inhibition impacts mitochondrial function. Our previous isothermal calorimetry analysis confirmed that both PES and PET-16 interact directly with purified human HSP70 [29]. X-ray crystallographic analysis of PET-16 with the HSP70 orthologue DnaK also revealed that it binds at an allosteric pocket within the C-terminus of this chaperone, and consequently inhibits substrate binding [29]. However, whether these compounds inhibit the ATPase activity of HSP70 had never been tested. ATP hydrolysis contributes to highly regulated allosteric changes within the HSP70 protein that are critical to its chaperone functions. To address this question, we purified human HSP70 protein produced in bacteria and assessed the ability of this protein to hydrolyze ATP. In this assay, purified HSP70 protein was able to hydrolyze a significant fraction of added ATP, while the addition of PES or PET-16 markedly inhibited this activity in a dose-dependent manner (Figure 5A). In contrast, the autophagy inhibitor chloroquine, which does not bind HSP70, had no effect on ATP levels (Figure 5A). We next tested the ability of PES and PET-16 to inhibit the proper folding and solubility of a well-characterized ‘client’ protein of HSP70, firefly luciferase [44]. Using the A875 melanoma cell line engineered to stably express the firefly luciferase gene, we found that expression of luciferase activity is notably decreased following incubation of these cells with PES and PET-16, but is not affected by exposure to the negative controls Cyclo-225 and TPPO (Figure 5B).

### Mitochondrial proteins exhibit altered expression patterns after HSP70 inhibition

Cytosolic HSP70 is an obligate co-chaperone of HSP90. Recent studies have shown that a portion of cellular HSP90 protein localizes to the mitochondria of tumor cells, but much less HSP90 is found in mitochondria of non-transformed cells. Tumor mitochondria also possess an organelle-specific member of the HSP90 family called TRAP-1 [16, 22, 23]. Several ‘client proteins’ of mitochondria-localized HSP90 and TRAP-1 have been identified, and these include cyclophilin D (CypD), hexokinase II (HKII), AKT (protein kinase B), SIRT3 and Survivin [13, 14, 16, 17]. CypD is a component of the permeability transition pore, HKII contributes to ATP production, and mitochondrial localized Survivin promotes cellular respiration. To address whether HSP70 inhibition also would affect these mitochondrial-localized proteins, we carried out western blot analysis on detergent-soluble lysates from several different tumor cell lines treated with PES and PET-16. This analysis revealed that the expression of all five of these proteins (CypD, HKII, AKT, SIRT3 and Survivin) were notably altered when assayed in whole cell extracts and isolated mitochondria of PES- and PET-16-treated cells (Figure 5C-5F). While there was a reduction in expression levels for CypD, HKII, AKT and SIRT3 (Figure 5C-5E), Survivin accumulated as aggregates (Figure 5F). Loss of protein forms from the soluble cell fraction often is accompanied by their accumulation in detergent insoluble fractions of cells treated with PES or PET-16, as we previously demonstrated [29, 30]. The notable reduction in the availability of these functionally important proteins at mitochondria resulting from HSP70 inhibition might be predicted to impact mitochondrial respiration. Therefore, we performed an analysis using a Seahorse bio-analyzer to measure the oxygen consumption rate (OCR) in cells. While various tumor cell lines exhibit different mitochondrial oxygen consumption profiles, as expected, the data show that treatment with PET-16 produces a marked decrease in OCR in all cases (Figure 5G).

**Figure 6:**
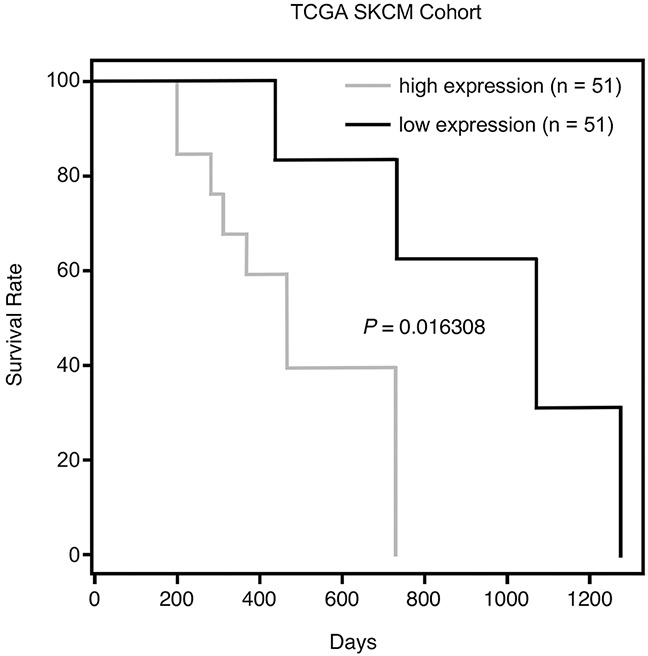
Kaplan-Meier survival data from TCGA SKCM Cohort Overall survival analysis of TCGA primary melanoma patients whose tumors were divided into two subgroups, having either high or low expression of PABPC1.

**Figure 7:**
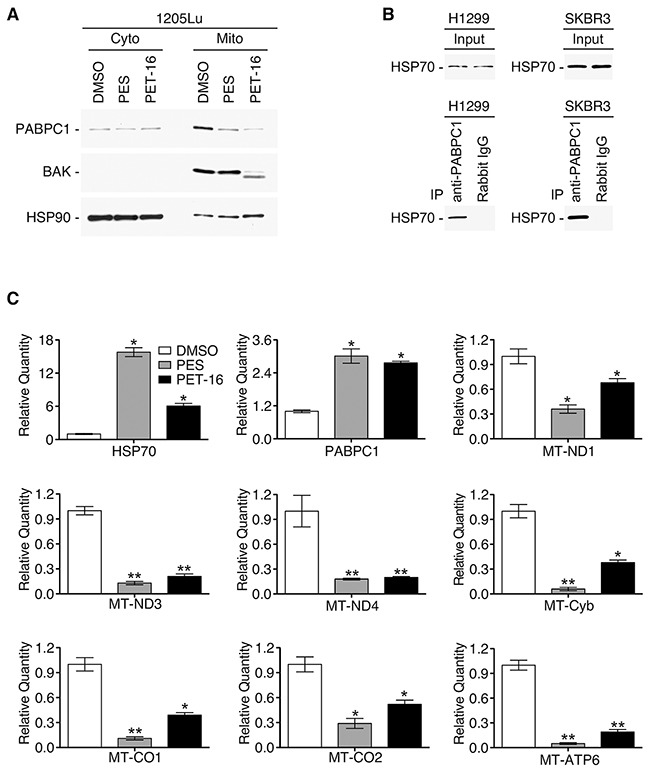
HSP70 inhibitors modify mitochondrial components **(A)** Western blot analysis of the PABPC1, BAK and HSP90 proteins in purified mitochondrial and cytoplasmic fractions of H1299 cells after 24 h of PES or PET-16 treatment (20 μM). **(B)** IP-WB analysis of the HSP70 and PABPC1 interaction in H1299 or SKBR3 breast carcinoma cells. **(C)** RT-qPCR analysis of RNA from A875 cells before and after treatment with PES or PET-16 (20 μM, 24 h) to assess abundance of nuclear encoded HSP70 and PABPC1 as well as several mitochondrial-encoded RNAs, as indicated. Values shown reflect mean±SD of technical triplicates. *P<0.05, **P<0.005.

We next sought to identify other mitochondrial client proteins of HSP70 whose expression is altered following HSP70 inhibition. Toward this goal we performed LC-MS/MS analysis of size fractionated mitochondrial proteins from untreated and PES-treated cells (Supplementary Figure 3). One of the proteins identified as being lost from mitochondria of PES-treated H1299 cells was polyadenylate binding protein 1 (PABPC1). Interestingly, our analysis of RNA sequencing data (RNA-seq) of patients with primary melanoma that are available from the TCGA database indicated that there is a significant association between the level of PABPC1 mRNA and overall survival (p=0.016), as presented in Figure 6. PABPC1 is an RNA binding protein that controls RNA stability and translation [45, 46]. This protein has a hypothesized, but still undefined, role at the mitochondria and our data indicate that high levels of PABPC1 expression in melanomas are associated with increased expression of genes encoding several mitochondrial proteins (Supplementary Figure 4). Western blot analysis confirmed that, following treatment with PES or PET-16, the level of PABPC1 was notably decreased in the mitochondrial fraction of H1299 lung carcinoma cells as well as in 1205Lu melanoma cells; in each case, there was a much more modest decrease in the abundance of cytosolic PABPC1 (Figure 7A). We next analyzed the interaction of HSP70 with PABPC1, which would support the premise that PABPC1 might be a ‘client’ of HSP70. Immunoprecipitation-western blot (IP-WB) analysis confirmed the interaction of these proteins (Figure 7B). Although the specific role of PABPC1 in mitochondria remains to be investigated, a recent study provided evidence that this RNA binding protein is able to interact with several mRNA species in mitochondria when specifically targeted to this organelle [46]. Given the reduced abundance of PABPC1 in mitochondria of cells treated with HSP70 inhibitors, we next analyzed the steady state level of several mitochondrial-encoded RNAs in the absence and presence of PES or PET-16. Quantitative reverse transcription-polymerase chain reaction analysis (RT-qPCR) of the levels of the mitochondrial electron transport components MT-ND1, MT-ND3, MT-ND4, MT-CO1, MT-CO2, MT-Cyb and MT-ATP6 indicated that all seven genes exhibit reduced RNA levels in PES- and PET-16-treated cells, relative to the control (Figure 7C). Taken together, our results provide evidence that HSP70 contributes in multiple ways to mitochondrial protein quality control in transformed cells. This new information now provides a molecular framework for the potential use of HSP70 inhibitors in the targeting of tumor versus normal mitochondria for cancer therapy.

## DISCUSSION

HSP70 is a key factor regulating cellular protein quality control, and is a cancer-critical survival protein [21–25]. It has a broad cellular distribution, and functions in multiple cellular compartments. Here we present evidence that the HSP70 protein also is abundantly present in mitochondria of tumor cells; however, it is generally undetectable or is present at quite low levels in mitochondria of unstressed, non-transformed, cells and tissues. This finding suggests that targeting the HSP70 proteostasis network will, both directly and indirectly, alter tumor mitochondria function, providing potential new avenues for anti-cancer treatment strategies. Such an approach may be especially attractive for cancers that do not respond, or have developed resistance, to more traditional or targeted therapies.

Using our novel class of HSP70 inhibitors, we determined that inhibiting HSP70 leads to notable mitochondrial dysfunction at several levels, including: disruption of mitochondrial structural integrity, loss of mitochondrial membrane potential, reduction in cellular ATP levels, impaired oxidative phosphorylation, and a multi-faceted inhibition of overall protein quality control. The latter is exemplified by alterations in the expression pattern and/or abundance of many mitochondrial proteins. Proteins whose expression pattern is altered in mitochondria following HSP70 inhibition include HKII and CypD; these proteins normally contribute to metabolic coupling of glycolysis and oxidative phosphorylation, as well as maintaining mitochondrial membrane integrity. Also, the scaffold-signaling protein p62 plays an important role in the triage of damaged, misfolded, or otherwise unneeded proteins for proteasomal degradation. Of particular interest, therefore, is our finding that treatment of cells with HSP70 inhibitors leads to altered p62 expression in mitochondria as well as an increased abundance of ubiquitin-bound proteins; both of these findings serve as reliable indicators of impaired proteostasis. Our investigation also identified PABPC1 as an HSP70 client protein present in mitochondria of tumor cells. PABPC1 is an RNA-binding protein that influences the stability and translation of many mRNAs, raising the possibility that PABPC1 may contribute to the import of specific transcripts into mitochondria or may play a role in the regulation of transcripts encoded by the mitochondrial genome. We found that exposure of cells to HSP70 inhibitors leads to a reduction in abundance of mitochondrial PABPC1, and this correlates with impaired mitochondrial function as well as reduced levels of several mitochondrial encoded RNAs.

Extensive differences have been identified between the functions of mitochondria in tumor cells relative to those in non-transformed cells [6–13]. These differences include tumor-related changes in energy production, altered production and tolerance of reactive oxygen species, and modulation of the mitochondrial permeability transition pore. Concomitantly, a large number of mitochondrial proteins and RNAs are qualitatively and/or quantitatively different in tumor cells versus their counterparts in non-transformed precursors. Among these are factors central to bioenergetics, metabolism, and antioxidant defense. Although mitochondria have their own genome, the vast majority of the ∼ 1,000-1,500 different proteins found in these organelles are encoded by nuclear genes that are imported from the cytosol. These proteins are potential clients for the mitochondrial protein quality control network. Appropriate crosstalk between mitochondria-dependent signaling pathways and cytosol/nuclear activities are needed to maintain the well-being of the cell and the organism. Previous work from our group has shown that the HSP70 molecular chaperone helps direct the mitochondrial trafficking, import, and integrity of many client proteins; thus, impairing HSP70 function can alter these diverse activities. For example, we have shown previously that PES reduces the HSP70-mediated mitochondrial trafficking of the tumor suppressors p53 and ARF, interfering with their roles in apoptosis and autophagy, respectively [27, 37].

Given the enhanced stress conditions associated with tumorigenesis, it is likely that tumor cell mitochondria have evolved heightened protein- and RNA-quality control networks to secure protein homeostasis and maintain cell survival. While the specific molecular determinants of mitochondrial quality-control systems are poorly understood, the results obtained in this investigation support the conclusion that the protective activities of the stress-induced HSP70 molecular chaperone are critical for maintaining the integrity and myriad functions of these organelles in tumor cells. Accordingly, HSP70 inhibitors represent a new class of potential anti-cancer agents, expected to have minimal deleterious effects in normal tissue.

## MATERIALS AND METHODS

### Reagents and antibodies

Sulfo-NHS-SS-Biotin (Biotin) and PES (2-Phenyl-ethynesulfonamide or Pifithrin-μ) were purchased from Thermo Fisher Scientific Inc. and EMD Millipore Corporation (Billerica, MA), respectively. The HSP70 antibodies used for the affinity purification studies were from Enzo Life Sciences, Inc. (Farmingdale, NY, USA). The other antibodies were from Cell Signaling Technology, Inc. (Danvers, MA, USA). Secondary antibodies conjugated to horseradish peroxidase were used at a dilution of 1:10,000 (Jackson ImmunoResearch Laboratories, Inc., West Grove, PA, USA).

### Mass spectrometry and electron microscopy

Liquid chromatography-tandem mass spectrometry was performed by the Genomics Institute and Abramson Cancer Center Proteomics Core Facility at the Perelman School of Medicine, University of Pennsylvania. Transmission electron microscopy (EM) and EM imaging were performed by The Electron Microscopy Resource Laboratory, Biomedical Research Core Facilities, at the Perelman School of Medicine, University of Pennsylvania.

### Proteins and PES-interactions

The human stress-inducible HSP70 (residues 1-641 was cloned into the pET25 vector (EMD Millipore Corporation, Billerica, MA USA) between the *NdeI* and *XhoI* restriction sites. The full-length HSP70 protein was produced with a N-terminal His_6_-tag in the *E. coli* BL21 Star (DE3) strain (Invitrogen catalog number C6010-03) and grown at 37°C in LB medium containing 50 μg/ml of carbenicillin (Sigma-Aldrich Co., St. Louis, MO, USA) as described previously [32]. Preparation of biotin-conjugated PES was described previously [31, 33, 34]. Gamitrinib (G-TPP) containing triphenylphosphonium was a generous gift from Dr. Dario Altieri, The Wistar Institute.

### Cell lines

All “WM” human melanoma cell lines were established and obtained from Dr. Meenhard Herlyn, The Wistar Institute, and their identity was confirmed by DNA fingerprinting. Other cell lines were purchased from American Type Culture Collection (ATCC, Manassas, VA, USA) and generally were used within six months of purchase. Cells typically were maintained in DMEM containing 10% FBS (GE Healthcare Life Sciences, SH30071.03) and 1% penicillin/streptomycin (GIBCO) at 37°C, except as follows: some melanoma cell lines were grown in RPMI 1640 (Life Technologies, 11875-085) supplemented with 10% fetal bovine serum (GE Healthcare Life Sciences, SH30071.03), 50 μg/mL gentamicin (Life Technologies, 15750060), and 25 mmol/L HEPES (Life Technologies, 15630080): primary human neonatal epidermal melanocytes (1° melanocytes) were isolated as described [47] and cultured in M254CF media (Invitrogen). A875 melanoma cells were engineered to stably express the firefly luciferase gene in the pWZL-Luciferase construct using Lipofectamine LTX (Life Technologies); stably expressing cells were selected in the presence of 75 μg/mL Hygromycin B.

### Subcellular fractionation and immunoblotting

Cellular cytosolic and mitochondrial fractions were purified as we previously detailed [48–50]. Immunoblotting (WB) and IP-WB analyses of cultured cell extracts and subcellular fractions were as we reported, and detergent (1% NP40)-soluble and detergent-insoluble cell fractions were prepared as detailed previously [27, 30]. All experiments with mice conformed to the guidelines of The Institutional Animal Care and Use Committee of the Perelman School of Medicine at the University of Pennsylvania, which approved all animal work according to federal and institutional policies and regulations.

### Mitochondrial membrane depolarization, cellular ATP depletion and luciferase assays

Changes in the mitochondrial membrane potential were measured using a Guava Mitochondrial Depolarization Assay (EMD Millipore) following manufacturer's recommendations. Total cellular ATP levels were measured using CellTiter-Glo assays (Promega). Values shown were normalized to total ATP abundance in control vehicle-treated cells. Error bars represent standard deviation (SD). Quantitation of luciferase activity was measured in a luminometer using the Luciferase Assay System (Promega).

### ATPase assays

Relative abundance of ATP was determined as previously described [51], in reaction conditions of 25 mM Citric Acid (pH 5.5), 10 mM MgCl_2_, and 20 mM KCl. The reaction was stopped with CellTiter-Glo Reagent (Promega), and luminescence was recorded using the Lumat luminometer. Measurements were performed in triplicate.

### Mitochondrial oxygen consumption rates (OCR)

Ten thousand melanoma cells were plated in Seahorse XFp cell culture miniplates for 16-18 hours before mitochondrial function assays. The cells were subsequently treated with PET-16 for 24 hours, and subjected to the Seahorse XF Cell Mito Stress Test, according to manufacturer's protocol. Briefly, cell medium was replaced with Seahorse XF Base Medium (supplemented with 100 mM Pyruvate, 200 mM Glutamine, and 2.5 M Glucose) and incubated in a non-CO_2_ incubator for one hour before the start of the assay. Basal OCR was measured using the Seahorse XFp Extracellular Flux analyzer. Measurements were performed after injection of three compounds affecting bioenergetics: 1 μM oligomycin (Seahorse Bioscience, North Billerica, MA), 1 μM carbonyl cyanide 4-(trifluoromethoxy)phenylhydrazone (FCCP) (Seahorse Bioscience) and 0.5 μM Rotenone/Antimycin A (Seahorse Bioscience). Upon completion of the Seahorse XFp Flux analysis, cells were lysed to calculate the protein concentration. The results were normalized to protein abundance in corresponding wells. Data are representative of three biological replicates.

### Bioinformatics analysis

The raw RNA-Seq data (BAM files) from The Cancer Genome Atlas (TCGA) database on SKCM samples were downloaded from the UCSC Cancer Genomics Browser. Read counts were summarized by using featureCounts [52] with the parameters that only paired-ended, not chimeric, and well mapped (mapping quality >= 20) reads were counted. Normalization was applied to eliminate bias from sequencing depths and gene lengths in edgeR [53], and RPKMs (Reads Per Kilobase of transcript per Million mapped reads) were obtained for every gene. Based on the RPKM values of PABPC1, the samples were separated into two groups: high and low, with median as the cutting point. Then differential expression (DE) analysis and Kaplan-Meier survival analysis were conducted to compare these two groups. The DE p values were transformed to z-scores, based on which we ranked all the genes.

## SUPPLEMENTARY MATERIALS FIGURES


