# Supplementary material for: Inhibition of stress-inducible HSP70 impairs mitochondrial proteostasis and function
Source: Oncotarget. 2017 Apr 21;8(28):45656–69. doi: 10.18632/oncotarget.17321 (PMC5542216; doi:10.18632/oncotarget.17321)
Supplement: Supplementary file 1 [file oncotarget-08-45656-s001.pdf]

Inhibition of stress-inducible HSP70 impairs mitochondrial proteostasis and function

SUPPLEMENTARY FIGURES

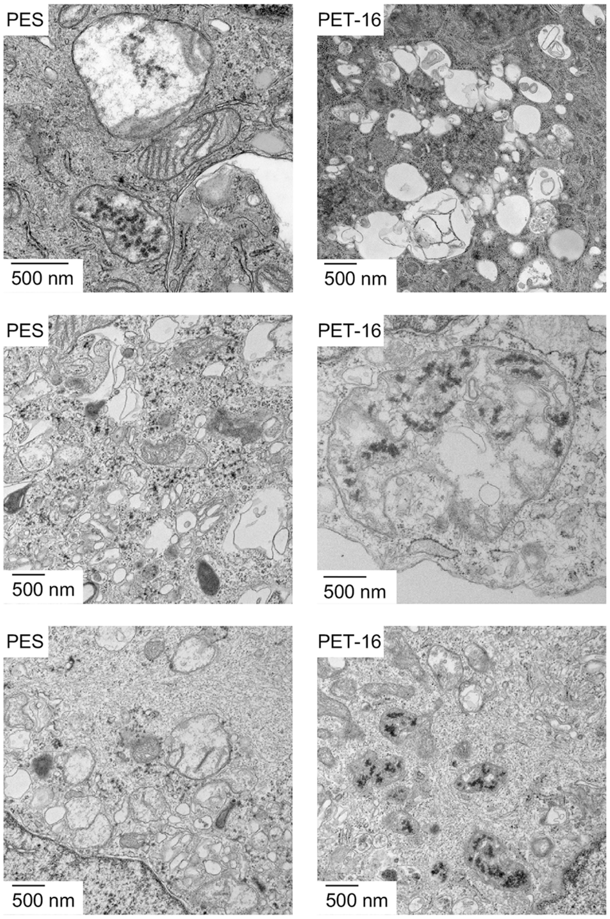

Supplementary Figure 1: Transmission electron micrographs (EM) of cells after treatment with HSP70 inhibitors. Representative images of A875 melanoma cells that were exposed to 20 µM PES or PET-16 for 24h.

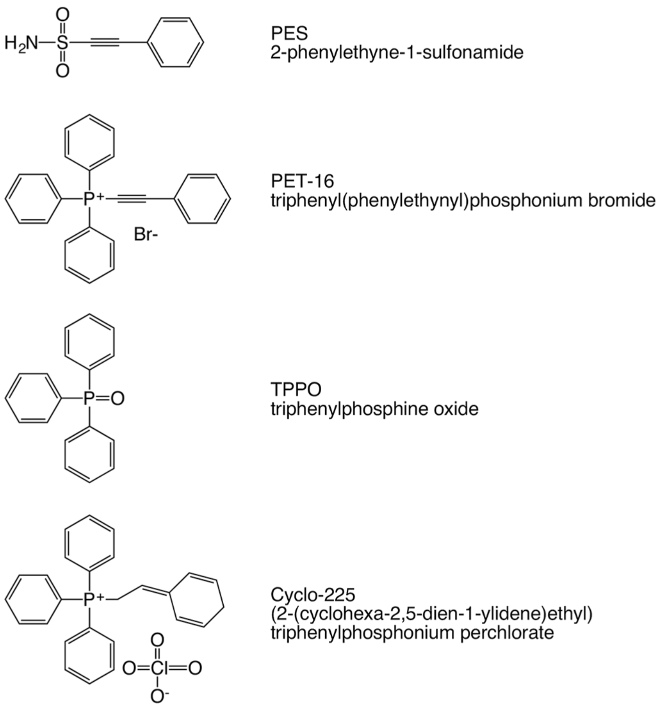

Supplementary Figure 2: Chemical structures of small molecules used in this study. Structures shown for PES, PET-16, TPPO and Cyclo-225.

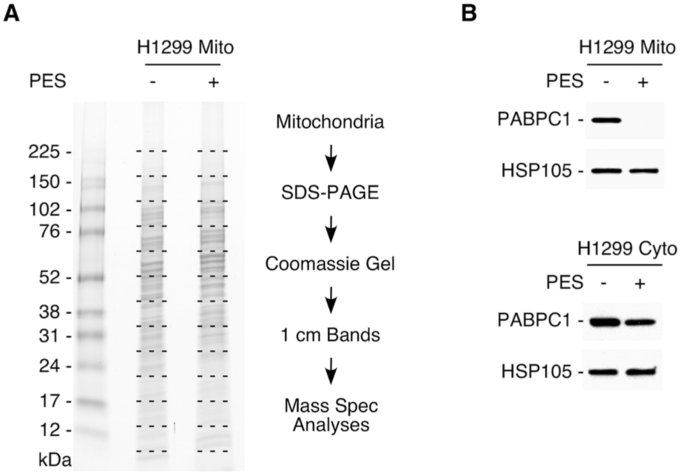

Supplementary Figure 3: PES alters expression pattern of mitochondrial proteins. (A) Isolated mitochondrial proteins from H1299 cells before and after PES treatment (20 µM, 24 h) were separated by SDS-PAGE followed by Coomassie staining and contiguous one-centimeter bands were isolated. Proteins subjected to nano-LC-MS/MS analysis and evaluated for changes in abundance of soluble proteins. **(B)** Western blot analysis of the PABPC1 and HSP105 proteins in purified mitochondrial and cytoplasmic fractions of H1299 cells after 24 h of PES treatment (20 µM).

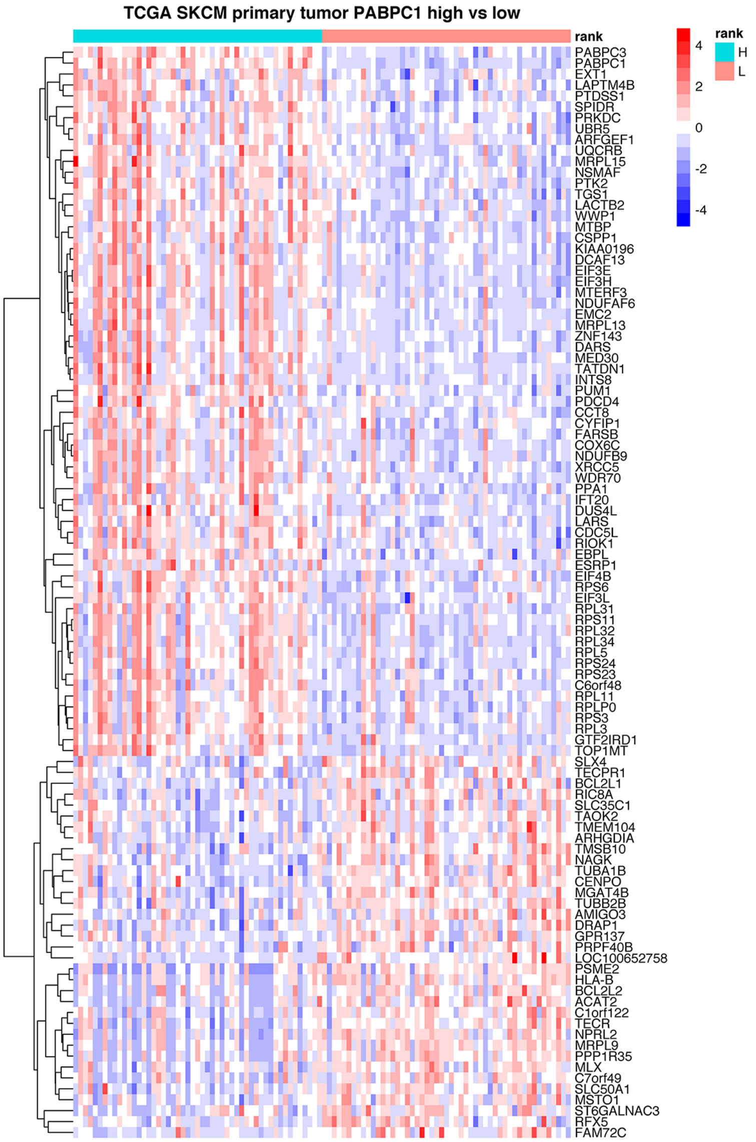

Supplementary Figure 4: Heat map of differentially expressed genes in TCGA melanoma patients. The data represent 100 significantly expressed genes that distinguish patients having high or low expression of PABPC1.
